# Annalist

**Published:** 1849-10

**Authors:** 


### ANNALIST.

The editor of the Annalist, Dr. N. S. Davis, having accepted the appointment of Professor of Physiology in the Rush Medical College, at Chicago, Illinois, that journal has been discontinued. Dr. Davis has had much to say heretofore concerning the "reforms" needed in Ameri can schools of medicine. It remains to be seen what will be the effect upon his mental optics of a more intimate relation to those institutions. We venture to hope that on a better acquaintance with them he will deem more charitably of their deserts.

